# Case of Persistant Vomiting, Etc.

**Published:** 1850-05

**Authors:** Austin Flint

**Affiliations:** One of the attending physicians at the Buffalo Hospital of the Sisters of Charity


ART. IV.—*Case of Persistent Vomiting and Purging, with Suppression of Urine. Sudden Death. Granular Degeneration of Kidneys.* Communicated by AUSTIN FLINT, M. D., one of the attending physicians at the Buffalo Hospital of the Sisters of Charity.

Mrs. Mary Cooncan, aged 35, married, (husband gone to California,) entered the hospital March 23d, 1850.

The record of the case, with the exception at the autopsical appearances, was made by Mr. S. Eastman, one of the resident students of the hospital.

*History.*—She says she was attacked yesterday morning with vomiting and diarrhœa. Dr. G. N. Burwell visited her, and prescribed remedies which afforded some relief, but the vomiting and diarrhœa, with pain, have continued more or less, up to the present time. She states that she had six or eight dejections during the night, containing blood and mucus. Has had no dejection for the last six hours. Vomited to-day after taking drink.

She says she has been in pretty good health prior to present illness.

*Present Symptoms.*—No appetite. No pain. Considerable thirst. Tongue moist, and covered with yellowish coating. Skin cool. Pulse 92, with some sharpness. Has slight cough and expectoration. Complains of a choking sensation. She has not menstruated for five weeks, and quantity heretofore, at mentstrual periods, quite small.

*Treatment.*—Sinapism to epigastrium. Creosote gtt. i after each act of vomiting. Enema of t. opi. ʒi. after second dejection.

At 5 P. M., of same day, (23d) reports a little better. Has vomited five or six times this P. M. Has had two dejections, thin, yellowish, containing a little blood, and a few mucous flakes. Enema administered two hours ago: no dejection since. Has no pain. Skin warm. Pulse 80, soft. Tongue same.

March 24.—Reports somewhat better. Passed an uncomfortable night; six or seven dejections, containing some blood and mucus. Occasional nausea and vomiting. Says she has passed no urine for two days. No tenderness on pressure over abdomen, nor distension in hypogastric region. Tongue continues the same. Skin warm and mellow. Pulse 84, soft.

General aspect improved. Complains of itching sensation over the whole body. Slight cough and expectoration. Character of the latter not ascertained. Anorexia. Moderate thirst.

*Treatment.*—Repeat sinapism to epigastrium. Sptts. ether nitrosus ʒi. three times. Enema s. morphiæ, gr. i. S. morphiæ, gr. ¼ per orem.

6 P. M.—Reports a little better. Has vomited occasionally through the day. Four dejections, somewhat bloody. Pulse 80, soft. Skin warm. Slight cough continues. Has passed no urine.

March 25.—Reports not so well. Complains of weakness. Passed a comfortable night. Three dejections, dark colored, slightly bloody. Passes no urine. Vomiting continues to occur occasionally. The matter vomited has a greenish color. Tongue moist, furred, and of a brown color. Skin of normal temperature. Pulse 76, soft, compressible. Anorexia. Not much thirst. Some tenderness on pressure over dorsal vertebrae. Menstrual flux commenced last evening.

*Treatment.*—Emp. vesicat, 6 x 6 to epigastrium. Cont. morphia, and sptts. ether nitrosus.

6 P. M.—Reports the same. Occasional vomiting. Two dejections of a dark color, and very offensive. Complains of great weakness. *Passes no urine.* Pulse 84, soft. Hiccough.

March 26.—Reports feeling very ill. Occasional vomiting through the night. Complains of general malaise. Pulse 76, soft. Two dejections.

March 27.—Reports not so well. Six or eight dejections during the night. Occasional vomiting. Tongue as before. Skin warm. Pulse 76, soft. *No urine.*

*Treatment.*—Let her have warm hip bath, and fomentations to hypogastrium. Milk and lime water, for diet.

P. M.—No material alteration in symptoms. Still no urine. No evidences of distended bladder, on examination of abdomen. She has an occasional desire to pass urine, but not great.

March 29.—Skin cool. Pulse the same. Respiration not labored nor accelerated. Aspect somewhat wild. Frequently asked for drink. Held ice in her mouth. Took some nourishment during the day, which was not rejected by the stomach. She had no vomiting or nausea during the day. She seemed extremity nervous, and almost hysterical. Learned that her son had been carried to the *pest house* with small pox, which appeared to occasion much anxiety. She was seen several times during the day, and no indications of impending dissolution observed; but, from the cessation of the vomiting and purging, the other symptoms remaining the same, there seemed to be an improvement. Still, no discharge of urine.

Death occurred at 5, P. M., without any change having been noticed by any one in the ward, until a moment before dissolution, when her respiration was observed to be short and gasping.

March 30th.—Autopsy, 1½, P. M. The funeral having been appointed at 3, P. M., there was time for only a cursory examination. This will account for the omission to extend the dissection as minutely as could have been desired.

*Chest first examined.*—No pleuritic adhesions. Lungs presented considerable emphysema. Dependent portions engorged, remainder more exsanguine than usual. No farther morbid appearances.

*Heart.*—Between one and two ounces of sanguinolent serosity in pericardial sac. No effusion of lymph, or other evidences of inflammation. Heart seemed moderately hypertrophied. Right ventricle distended with blood, mostly fluid, with some dark, loose coagula. Left ventricle nearly empty, no coagula. Right auricle much distended. Left auricle nearly empty. Sigmoid and auriculo-ventricular valves normal.

*Abdomen.*—Intestines moderately tympanitic. External appearance not morbid. Internally not examined. No effusion into peritoneal sac observed. Liver, on cursory examination, seemed not morbid. Bladder perfectly empty, and contracted as in epidemic cholera.

Both kidneys presented extensive granular degeneration, the morbid deposit supplanting the proper tissue almost entirely in the cortied portion, encroaching on the tubuli, and, in several instances, completely surrounding and insulating the latter. In size the organs did not appear to deviate from the average. External surface smooth, not tuberculated. The right kidney was congested, the left exsanguine. The right kidney presented an ecchymosed appearance over about one quarter of its exterior surface,

*Head.*—Increased sub-arachnoidean effusion. No effusion of fibrin. One and a half ounces (as estimated) of serum, slightly turbid, at the base of the brain, in the arachnoid sac. Lateral ventricles nearly empty. The brain presented remarkable firmness, as if it had been hardened by immersion in alcohol. Color normal. Very few red points on section. No other morbid appearances. The body presented moderate embonpoint. No oldema of face, nor of lower extremeties.

*Remarks.*—The symptoms presented in this case at first, and, indeed, during its progress, did not lead to a correct diagnosis, nor to an anticipation of the termination. The persistence of the vomiting and purging occasioned surprise, but there did not exist, in connection therewith, local and general symptoms pointing to inflammation of the gastro-intestinal tube, nor to the existence of lesions which would be expected to lead to a fatal result. The reader may think that the suppression of urine should have created more apprehension of danger than was entertained. With respect to this symptom, the evidence of its existence rested on the statement of the patient; and when it was ascertained that the bladder was not distended, there seemed to be room for suspicion that either the patient deceived herself on this point, or, from a peculiar morbid perverseness which, as is well known, is occasionally met with, made erroneous statements. The absence of the symptoms usually attending suppression of the urinary secretion (coma more especially), when, as appeared from the patient's statements, this suppression had continued for several days in succession, were considered to afford sufficient ground for doubting the fact. On the day on which the sudden death occurred, the symptoms, irrespective of the suppression of urine, appeared to indicate improvement. The nervous system, it is true, exhibited marked disorder on this day, but not in a way to excite alarm; on the contrary, it was calculated rather to allay apprehension, from its resemblance to hystria, and to favor the hypothesis with respect to the secretion of urine. The sudden termination, therefore, was quite unlooked for. It is freely confessed that the facts disclosed by the cursory examination after death were not less unexpected than the progress and result of the case. The chest was first examined because it was anticipated that the immediate cause of death would be there discovered, and to this object the autopsy was necessarily, in a great measure, limited. It was suspected that an anlusismal tumour might be found, or the heart itself might have been the seat of rupture.

The appearance of the bladder showed conclusively that it had been empty for some time previous to death; and the condition of the kidneys afforded an explanation of the non-secretion of urine. The granular deposit was so extensive as to have caused the absorption of nearly the whole secreting tissue of the organs. After an examination of the kidneys, it was easier to understand why the urinary secretion should have been suppressed, than to account for the fact that this event should have taken place so suddenly. It is an extraordinary feature of the case, that the patient should have been attacked while in tolerable health, and that no evidence of the existence of granular degeneration of the kidneys appears in her previous history in so far as this has been ascertained. At the time of her seizure, she was engaged in the duties of a house servant. What the symptoms were, denoting impaired health, is not known; but it does not appear that she had anasarea, or dropsical effusion into any of the serous cavities. Yet, it is by no means probable that the whole amount of the granular degeneration of the kidneys occurred at the instant of the attack, or in a very short space of time previously. The affection is essentially chronic in its course in the great majority of cases, but we may conceive that, like tuberculosis, in some rare instances, the deposit may take place with great rapidity. Under the supposition that more or less of the renal affection must have existed for some time previous to the fatal attack, it may be asked, why were not the usual effects distinctly present, more especially dropsy? In view of this inquiry, it is to be recollected that, although dropsical effusion occurs in connection with this affection in the vast majority of instances, does not necessarily follow. Cases have occurred of extensive granular degeneration without any dropsical effusion.

Another peculiar, and highly interesting feature of this case is the exemption from the usual characteristic symptoms, or effects of suppression of urine, and the consequent retention in the blood of the urinary elements. Why were these symptoms, or effects, absent? The most rational explanation would seem to be, that the vomiting and purging performed a vicarious office, eliminating from the blood the poisonous principles which should have been excreted by the kidneys. In connection with this explanation, it is to be observed, that the sudden death of the patient was preceded by cessation of the vomiting and purging, and then were developed restlessness and nervous excitement which were thought to be hysterical. Under these circumstances, it would be expected that death would be preceded by coma. This, it may be reasonably supposed, would have been the case, had not effusion taken place into the arachnoid sac, to which, probably was due, in a great measure, the sudden termination, resulting from a suspension of asphyxia, the instinctive sense of the necessity of respiration. Effusion, with or without inflammation of some one or more of the serous structures, belong, as is well known, among the usual concomitants of granular disease of the kidneys, and in this instance the arachnoid was the serous structure preferred.

The remarkable firmness of the brain constitutes another interesting fact in the history of this case. We do not remember to have seen this noticed as incident to retention of urea in the blood, nor are we prepared to say what importance is to be attached to it in the present instance.

April 4, 1850.

